# Protein Expression of ZEB2 in Renal Cell Carcinoma and Its Prognostic Significance in Patient Survival

**DOI:** 10.1371/journal.pone.0062558

**Published:** 2013-05-02

**Authors:** Yong Fang, Jinhuan Wei, Jiazheng Cao, Hongwei Zhao, Bing Liao, Shaopeng Qiu, Daohu Wang, Junhang Luo, Wei Chen

**Affiliations:** 1 Department of Urology, First Affiliated Hospital, Sun Yat-Sen University, Guangzhou, China; 2 Department of Urology, Affiliated Jiangmen Hospital, Sun Yat-Sen University, Jiangmen, China; 3 Department of Urology, Affiliated Yantai Yuhuangding Hospital, Qingdao University Medical College, Yantai, China; 4 Department of Pathology, First Affiliated Hospital, Sun Yat-Sen University, Guangzhou, China; National Cancer Institute, National Institutes of Health, United States of America

## Abstract

**Background:**

ZEB2 has been reportedly shown to mediate the epithelial-to-mesenchymal transition (EMT) and disease aggressiveness in human tumors. However, the expression status of ZEB2 in renal cell carcinoma (RCC) and ZEB2’s clinicopathologic/prognostic significance are poorly understood.

**Methodology/Principal Findings:**

In this study, tissue microarray, immunohistochemistry (IHC) and western blot analyses were utilized to investigate the ZEB2 expression status in RCC and adjacent renal tissue samples. In our study, samples from 116 RCC patients treated with radical nephrectomy were used as a training set to generate a ZEB2 optimal cut-point for patient outcome by receiver operating characteristic (ROC) analysis. For validation, the correlation of ZEB2 expression with the clinical characteristics and patient outcomes in another set (including 113 patients) was analyzed to validate the obtained cut-point. In the training and validation sets, high expression of ZEB2, defined by ROC analysis, predicted a poorer overall survival and progression-free survival, as evidenced by the univariate and multivariate analyses. In different subsets of overall patients, ZEB2 expression was also a prognostic indicator in patients with stage I/II, stage III/IV, grade 1/2 and grade 3/4 disease (*P*<0.05). Downregulation of ZEB2 by shRNA decreased the migration and invasion ability of 769-P cells in vitro. Furthermore, high ZEB2 expression was positively correlated with vimentin expression and inversely linked to E-cadherin expression in RCC.

**Conclusions/Significance:**

Our findings provide a basis for the concept that high ZEB2 expression in RCC may be important in the acquisition of an aggressive phenotype. This evidence suggests that ZEB2 overexpression (examined by IHC) is an independent biomarker for the poor prognosis of patients with RCC.

## Introduction

Renal cell carcinoma (RCC) is the most lethal common urologic cancer, accounting for 2–3% of all cancers in adults [1], [2]. Surgical resection is the mainstay of treatment. However, more than 40% patients with RCC develop metastases after radical nephrectomy, and the 10-year cancer-specific survival rate is dismal [3], [4]. Biomarkers that could identify the recurrence potential of RCC may shape appropriate therapeutic strategies earlier in the course of this malignancy. Therefore, many groups studying RCC have focused on the discovery of promising molecular markers that are associated with the progression of RCC. However, the search for and identification of specific molecular and/or genetic alterations in RCC cells that have clinical/prognostic significance remains substantially limited.

The epithelial-mesenchymal transition (EMT) is a genetic program that controls cell migration in embryonic development and adult tissue homeostasis [5], [6]. The uncontrolled activation of EMT programs occurs in epithelial tumor cells and contributes to the formation of cancer stem cells and metastasis [6], [7], [8]. Zinc-finger enhance binding (ZEB) transcription factors ZEB1 and ZEB2 are crucial EMT activators [9]. The ZEB2 protein contains a central homeodomain, CtBP-binding and Smad-interacting domains and two zinc finger clusters at either end [10], [11]. ZEB2 directly binds to proximal E-boxes within the E-cadherin gene (*cdh1*) promoter and mediates transcriptional repression [11], [12]. Transcriptional repression is mediated through the relationship with the corepressor CtBP. However, this interaction is dispensable, at least for the attenuation of cdh1 transcription [12], [13]. ZEB2 was identified as a binding partner of R-Smads and was also shown to be part of the TGF-β pathway, which is involved in carcinogenesis [11]. In the context of regulating vimentin, ZEB2 observed in the EMT was associated with breast cancer cell migration [14]. The relevance of the ZEB2 protein to cancer progression has been investigated in various human tumors. Sayan et al. found that ZEB2 overexpression was an independent prognostic factor in bladder cancer and positively correlated with a poor therapeutic outcome [15]. The correlations between a high ZEB2/E-cadherin ratio and an aggressive phenotype and poor prognosis were observed in breast and ovarian cancers [16]. Additionally, Sakamoto et al. revealed that the overexpression of SIP1 and loss of E-cadherin were significantly correlated with delayed neck metastasis in oral tongue squamous cell carcinoma [17]. In addition, ZEB2 mediated the hypoxia-inducible factor 1 alpha-dependent repression of E-cadherin in von Hippel-Lindau tumor suppressor-null renal cell carcinomas [18]. However, the role of ZEB2 expression in localized RCC has not been fully elucidated.

The aim of our study was to evaluate whether ZEB2 plays a role in the development of RCC and to determine its prognostic significance. In this study, western blot, immunohistochemistry (IHC) and tissue microarray (TMA) analyses were utilized to investigate the expression levels of ZEB2 in RCC tissues. The ZEB2 IHC staining results were then correlated with patient survival rates using various statistical analyses. Next, we applied shRNA to knockdown ZEB2 in a RCC cell line to examine its effects on cell migration and invasion and to explore the potential mechanism by which ZEB2 regulates EMT in RCC tissues.

## Patients and Methods

### Ethics statement

The study was approved by the Institute Research Medical Ethics Committee of the Sun Yat-Sen University. No informed consent (written or verbal) was obtained for the use of the retrospective tissue samples from the patients within this study, most of whom were deceased. Informed consent was not deemed necessary by the Ethics Committee, who waived the need for consent. All of the samples were anonymous.

### Patients and tissue specimens

We randomly collected 229 patients with unilateral, sporadic RCC treated with radical nephrectomy between May 2000 and July 2007 at the First Affiliated Hospital and Cancer Center of Sun Yat-Sen University, Guangzhou, China. The patients were randomly separated into training and validation cohorts. For patient selection, histological proof of RCC was required. None of these patients received adjuvant therapy. The TNM 2009 staging system was utilized to classify RCC patients [19]. Of these patients, 104 patients were stage I, 47 patients were stage II, 47 patients were stage III and 31 patients were stage IV. The grading system used in the study followed the Fuhrman four-grade scale. The histological subtypes were classified in accordance with the 2002 AJCC/UICC classification system, and only tumors of clear-cell, papillary, and chromophobe histology were included in this study. Patients were excluded if they had a history of preoperative anticancer treatment or did not have complete follow-up data available. The overall survival was measured from the date of the last follow-up visit for survivors. The median follow-up was 25.37 months (range, 0.57 to 101.83 months). The institutional review board at each participating institution approved the retrospective analysis of anonymous data.

### Western blotting analysis

Equal amounts of whole tissue lysates were resolved by SDS-polyacrylamide gel electrophoresis (PAGE) and electrotransferred onto a polyvinylidene difluoride (PVDF) membrane (Pall Corp., Port Washington, NY, USA). Subsequently, the lysates were incubated with a primary anti-ZEB2 antibody (Cat. No. HPA003456, Sigma, St. Louis, MO, USA, 1∶1000 dilution). The immunoreactive signals were detected using the enhanced chemiluminescence kit (Amersham Biosciences, Uppsala, Sweden). The procedures were conducted according to the manufacturer’s instructions.

### Tissue microarray (TMA) construction

A tissue microarray was constructed as described previously [20]. In brief, formalin-fixed, paraffin-embedded tissue blocks and the corresponding H&E-stained slides were overlaid for TMA sampling. The slides were reviewed by a senior pathologist (B Liao) to determine and mark the representative tumor areas. Triplicates of 0.6-mm diameter cylinders were punched from the representative tumor areas of the individual donor tissue block and re-embedded into a recipient paraffin block at a defined position using a tissue arraying instrument (Beecher Instruments, Silver Spring, MD, USA).

### Immunohistochemical analysis

IHC studies were performed using a standard streptavidin-biotin-peroxidase complex method [21]. In brief, the tissue sections were deparaffinized and rehydrated. Endogenous peroxidase activity was blocked with 0.3% hydrogen peroxide for 20 min. For antigen retrieval, the slides were microwave treated and boiled in 10 mM citrate buffer (pH 6.0) for 10 min. Nonspecific binding was blocked with 10% normal goat serum for 20 min. The slides were incubated with a rabbit polyclonal anti-ZEB2 (Cat. No. HPA003456, Sigma, St. Louis, MO, USA, 1∶100 dilution), anti-E-cadherin (Dako, Glostrup, Denmark, 1∶50 dilution) and anti-vimentin antibodies (Dako, Glostrup, Denmark, 1∶200 dilution) overnight at 4°C in a humidified chamber. The slides were then sequentially incubated with a secondary antibody (Envision; Dako, Glostrup, Denmark) for 1 hour at room temperature using 3′-3′ diaminobenzidine as a chromogen substrate. The nucleus was counterstained using Meyer’s hematoxylin. A negative control was obtained by replacing the primary antibody with normal rabbit or murine IgG. Known immunostaining-positive renal cancer slides were used as the positive controls.

### IHC evaluation

Cytoplasmic and nuclear immunoreactivity for ZEB2 was scored in a semi-quantitative method by assessing the number of positive tumor cells over the total number of tumor cells. The cores were assigned using 5% increments (i.e., 0%, 5%, 10% …100%) (Figure S1, Table S1) [22]. ZEB2 expression was assessed by 2 independent pathologists who were blinded to clinicopathological data. The data are expressed as the mean value of the triplicate experiments.

### Selection of cutoff score

ROC curve analysis was used to determine the cutoff value for ZEB2 expression in the training set using the 0,1-criterion, as described previously [20]. In the ZEB2 score, the sensitivity and specificity for each outcome under the study was plotted, thus generating various ROC curves. The score was selected as the cutoff value, which was closest to the points of maximum sensitivity and maximum specificity. The tumors designated as “low expression” for ZEB2 were those with scores below or equal to the cutoff value. Meanwhile, “high expression” tumors were those with scores above the value. To perform the ROC curve analysis, the clinicopathologic features were classified according to the following: age (≤mean age or >mean age), size (≤mean size or >mean size), TNM stage (I/II or III/IV), Fuhrman grade (G1/2 or G3/4), histological subtype (clear or papillary/chromophobe) and survival status (death due to RCC or censored).

### Cell culture and RNA interference

Human RCC 769-P cells (http://www.atcc.org) were cultured in RPMI-1640 medium supplemented with 10% fetal bovine serum. Short interfering RNA specifically against the *ZEB2* gene and the corresponding scrambled shRNA (Ambion, Austin, TX, USA) were transfected into the 769-P cells in 6-well plates using the Lipofectamine 2000 Reagent, according to the manufacturer’s instructions. pGPU6/GFP/Neo-shRNA plasmid targeting ZEB2 (shZEB2) was constructed by Shanghai JIMA Biologic Co., China. The ZEB2-specific shRNA sequence was as follows: sense 5-CACCGCATGTATGCATGTGACTTATTTCAAGAGAATAAGTCACATGCATACATGCTTTTTTG-3; antisense 5-GATCCAAAAAAGCATGTATGCATGTGACTTATTCTCTTGAAATAAGTCACATGCATACATGC-3. The gene silencing effect was measured by western blotting at 48 hours post transfection.

### Cell migration and cell invasion assay

The RCC cells were plated in 6-well plates and allowed to grow to confluence. For the cell migration assay, the medium was discarded and wounds were introduced by scraping the confluent cell cultures with a 10-µl pipette tip. The floating cells were carefully removed before the complete medium was added. The cells were incubated at 37°C in a humidified atmosphere of 95% air and 5% CO2. The wound healing process was monitored under an inverted light microscope (Nikon, Tokyo, Japan). The transwell cell invasion assay was performed in BD BioCoat Matrigel Invasion Chambers (Becton Dickinson Labware, Franklin Lakes, NJ, USA) with 8 µm porosity, according to the manufacturer’s instructions, for 48 hours. The experiments were performed 3 times.

### Statistical analysis

The statistical analyses were performed using the SPSS software program (SPSS Standard version 13.0, SPSS Inc, Chicago, IL, USA). ROC analysis was utilized to define the cutoff score for high ZEB2 expression by the 0,1-criterion, and the areas under curves (AUCs) were then calculated. The correlations between ZEB2 expression, EMT marker expression and clinicopathologic features were analyzed using the Spearman rank test. The statistical significance of the correlation between ZEB2 expression and metastasis-free survival was estimated using the log-rank test. Multiple Cox proportional hazards regressions were performed to identify the independent factors that had a significant impact on patient survival. The independent t test was performed to analyze the statistical significance between two preselected groups. A difference was considered significant if the *P* value from a two-tailed test was less than 0.05.

## Results

### The expression levels of ZEB2 in renal tissues by western blotting

**Figure 1:**
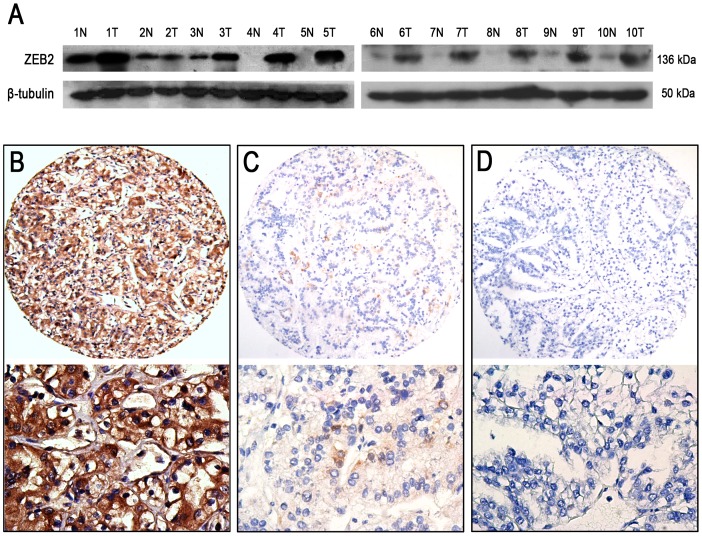
Expression of the ZEB2 protein in RCC and adjacent non-malignant renal tissues. (A) Up-regulated expression of the ZEB2 protein was observed in 8/10 RCC cases using western blot when compared to the adjacent non-malignant renal tissues. *T,* RCC tissue; *N,* non-neoplastic renal tissue. (B) High ZEB2 expression was observed in an RCC sample (case 63), in which more than 90% of the tumor cells revealed positive immunostaining of ZEB2 in the cytoplasm and nuclei (*upper panel*, ×100). (C) A RCC case (case 56) demonstrated low ZEB2 expression, in which fewer than 50% of the tumor cells showed immunoreactivity of the ZEB2 protein (*upper panel*, ×100). (D) Nearly negative expression of the ZEB2 protein was demonstrated in an RCC case (case 72, *upper panel*, ×100). The *lower panels* indicate the higher magnification (×400) from the area of the boxes in (B), (C) and (D), respectively.

To investigate the ZEB2 expression levels in RCC, we examined ZEB2 protein expression in 10 pairs of primary RCC and matched adjacent renal tissues by western blot. Our results showed that up-regulated expression of ZEB2 was detected in the majority of primary RCC tissue samples compared to the adjacent non-neoplastic renal tissues (Figure 1A).

### Selection of the cutoff value for ZEB2 IHC expression

**Figure 2:**
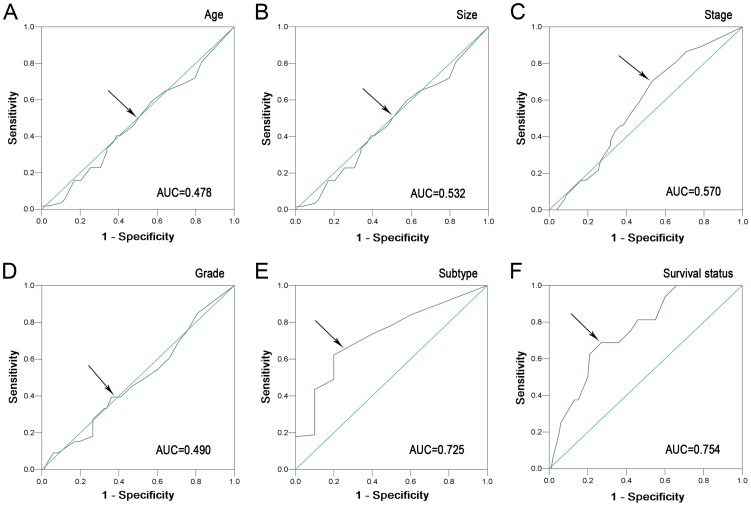
Receiver operating characteristic curves were used to determine the cutoff score for high ZEB2 expression in RCC. The sensitivity and specificity for each outcome were plotted, and the areas under curve (AUCs) were indicated: age (*P* = 0.683), tumor size (*P* = 0.561), TNM stage (*P* = 0.227), Fuhrman grade (*P* = 0.869), tumor subtype (*P* = 0.019) and survival status (*P* = 0.001).

ZEB2 IHC staining was detected in the cytoplasm and/or nuclei of tumor cells (Figures 1B–1D). To develop an optimal ZEB2 cutoff score for further analysis, we subjected the ZEB2 score of the training cohort to ROC curve analysis with respect to the clinical characteristics. The ROC curves for each clinicopathologic feature clearly showed the point on the curve closest to (0.0, 1.0), which maximizes both the sensitivity and specificity for the outcome, as described in a previous study [23]. Cancers with scores above the obtained cutoff value were considered to have high ZEB2 expression, which led to the greatest number of cancers classified, based on the presence or absence of a clinical outcome. As shown in Figure 2, the AUC for survival status had the biggest area. Based on this outcome, we selected a ZEB2 expression score of 55% as the optimal cut-point for survival analysis.

### Relationship between ZEB2 expression and RCC patient clinicopathologic features and survival

**Figure 3:**
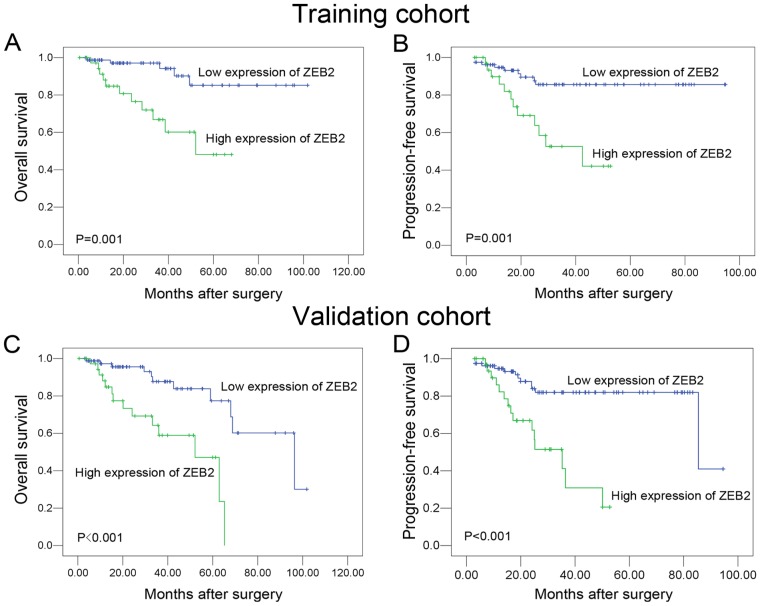
Kaplan-Meier survival analysis of ZEB2 expression in the training and validation cohort of patients with RCC (log-rank test). Kaplan-Meier survival analysis of ZEB2 expression for overall survival (A) and progression-free survival (B) in the training cohort (log-rank test). Kaplan-Meier survival analysis of ZEB2 expression for overall survival (C) and progression-free survival (D) in the validation cohort (log-rank test).

In the training cohort, high ZEB2 expression was observed in 38/116 (32.8%) of the RCC samples. A correlation analysis showed that there was no significant correlation between ZEB2 expression and the clinicopathologic features, including patient gender, age, tumor size, clinical stage, Fuhrman grade and histological subtype (*P*>0.05, Table 1). A Cox proportional hazards regression analysis demonstrated a significant impact of prognostic features (i.e., tumor size, TNM stage, Fuhrman grade and ZEB2 expression) on the patient survival rates (*P*<0.05, Table 2). Furthermore, a Kaplan-Meier analysis showed that ZEB2 was a powerful prognostic factor for overall survival (OS) and progression-free survival (PFS) (Figures 3A and 3B).

**Table 1:** The correlation between ZEB2 expression and clinicopathological features of patient with RCC.

	ZEB2 protein					
Variable	Training cohort			Validation cohort		
	All cases	High expression	*P* value*	All cases	High expression	*P* value*
Gender			0.256			0.497
Male	74	27 (36.5%)		78	25 (32.1%)	
Female	42	11 (26.2%)		35	9 (25.7%)	
Age (years)			0.790			0.134
≤51.7^†^	59	20 (33.9%)		52	12 (23.1%)	
>51.7	57	18 (31.6%)		61	22 (36.1%)	
Size (cm)			0.294			0.153
≤6.7^‡^	69	20 (29.0%)		65	23 (35.4%)	
>6.7	47	18 (38.3%)		48	11 (22.9%)	
TNM stage			0.709			0.047
I/II	79	25 (31.6%)		72	17 (23.6%)	
III/IV	37	13 (35.1%)		41	17 (41.5%)	
Fuhrman grade			0.967			0.062
G1/2	83	27 (32.5%)		86	22 (25.6%)	
G3/4	33	11 (33.3%)		27	12 (44.4%)	
Histological subtype			0.109			0.859
Clear	106	37 (34.9%)		107	32 (29.9%)	
Papillary/chromophobe	10	1 (10.0%)		6	2 (33.3%)	

* Chi-square test; ^†^Mean age; ^‡^Mean size.

**Table 2:** Univariate analysis of different prognostic factors in patients with RCC overall survival.

Variable	Training cohort			Validation cohort		
	All cases	HR (95% CI)	*P* value	All cases	HR (95% CI)	*P* value
Gender			0.567			0.742
Male	74	1.0		78	1.0	
Female	42	0.749 (0.279–2.014)		35	0.858 (0.346–2.127)	
Age (years)			0.119			0.892
≤54.0*	59	1.0		52	1.0	
>54.0	57	0.431 (0.149–1.242)		61	0.942 (0.396–2.238)	
Size (cm)			0.040			0.405
≤6.7^†^	69	1.0		65	1.0	
>6.7	47	1.319 (1.012–1.719)		48	1.097 (0.883–1.362)	
TNM stage			0.003			0.0004
I/II	79	1.0		72	1.0	
III/IV	37	4.767(1.699–13.372)		41	20.810 (4.840–89.476)	
Fuhrman grade			0.004			0.002
G1/2	83	1.0		86	1.0	
G3/4	33	4.500 (1.627–12.444)		27	3.987 (1.691–9.402)	
Histological subtype			0.879			0.969
Clear	106	0.854 (0.112–6.522)		107	1.040 (0.139–7.763)	
Papillary/chromophobe	10	1.0		6	1.0	
ZEB2 expression			0.002			0.002
Low	78	1.0		79	1.0	
High	38	5.344 (1.852–15.417)		34	4.031 (1.691–9.786)	

* Mean age; ^†^Mean Size; HR, hazards ratio; CI, confidence interval.

In the validation cohort, high ZEB2 expression was found in 34/113 (30.1%) of RCC cases. As shown in Table 1, a significant correlation was observed between ZEB2 expression and the clinical stage. Similar to the observations in the testing cohort, the high expression of ZEB2 was linked closely to a poorer OS and PFS in RCC patients (Figures 3C and 3D).

**Figure 4:**
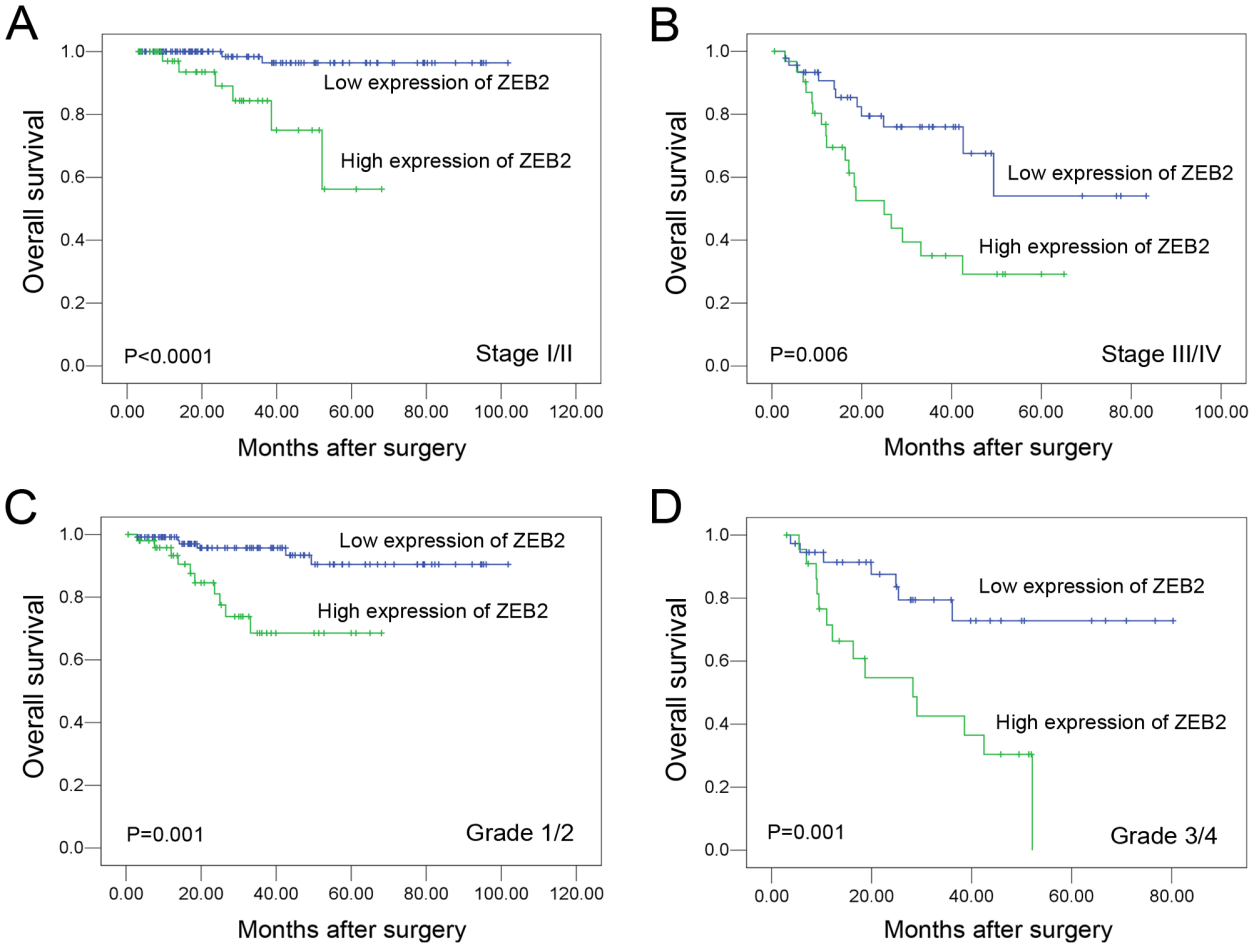
Kaplan-Meier survival analysis of ZEB2 expression in different subsets of overall patients with RCC (log-rank test). (A) *Stage I/II*, the probability of survival of stage I/II patients with RCC: low expression, n = 109; high expression, n = 42. (B) *Stage III/IV*, probability of survival of stage III/IV patients with RCC: low expression, n = 46; high expression, n = 32. (C) *Grade 1/2*, the probability of survival of grade 1/2 patients with RCC: low expression, n = 118; high expression, n = 51. (D) *Grade 3/4*, probability of survival of grade 3/4 patients with RCC: low expression, n = 37; high expression, n = 23.

Further survival analysis was performed with regard to ZEB2 expression in subsets of patients with different stages and grades. Our results demonstrated that high ZEB2 expression was a prognostic factor in RCC patients with stage I/II, stage III/IV, grade 1/2 and G3/4 (*P*<0.05, Figure 4).

### Independent prognostic factors for RCC

Because the variables that were observed to have a prognostic influence on RCC patients by the univariate analysis may correlate, the expression of ZEB2, TNM stage and Fuhrman grade, which were significant in the univariate analyses in both cohorts, were further evaluated in multivariate analysis. ZEB2 expression in RCC tissues was found to be an independent prognostic factor for poor overall survival in the training and validation cohorts (Table 3). In addition, the TNM stage and Fuhrman grade (*P*<0.05) were also evaluated as independent prognostic factors for patient overall survival in both cohorts (Table 3).

**Table 3:** Multivariate analysis of different prognostic factors in 229 patients with RCC.

Variable	Training cohort			Validation cohort		
	All cases	HR (95% CI)	*P* value	All cases	HR (95% CI)	*P* value
TNM stage			0.008			0.010
I/II	79	1.0		72	1.0	
III/IV	37	4.053 (1.441–11.405)		41	7.933 (1.640–38.361)	
Fuhrman grade			0.005			0.037
G1/2	83	1.0		86	1.0	
G3/4	33	4.319 (1.539–12.116)		27	3.491 (1.082–7.268)	
ZEB2 expression			0.007			0.033
Low	78	1.0		79	1.0	
High	38	4.392 (1.502–12.846)		34	3.561 (1.124–8.138)	

HR, hazards ratio; CI, confidence interval.

### ZEB2 regulates the migration and invasion of 769-P cells in vitro

**Figure 5:**
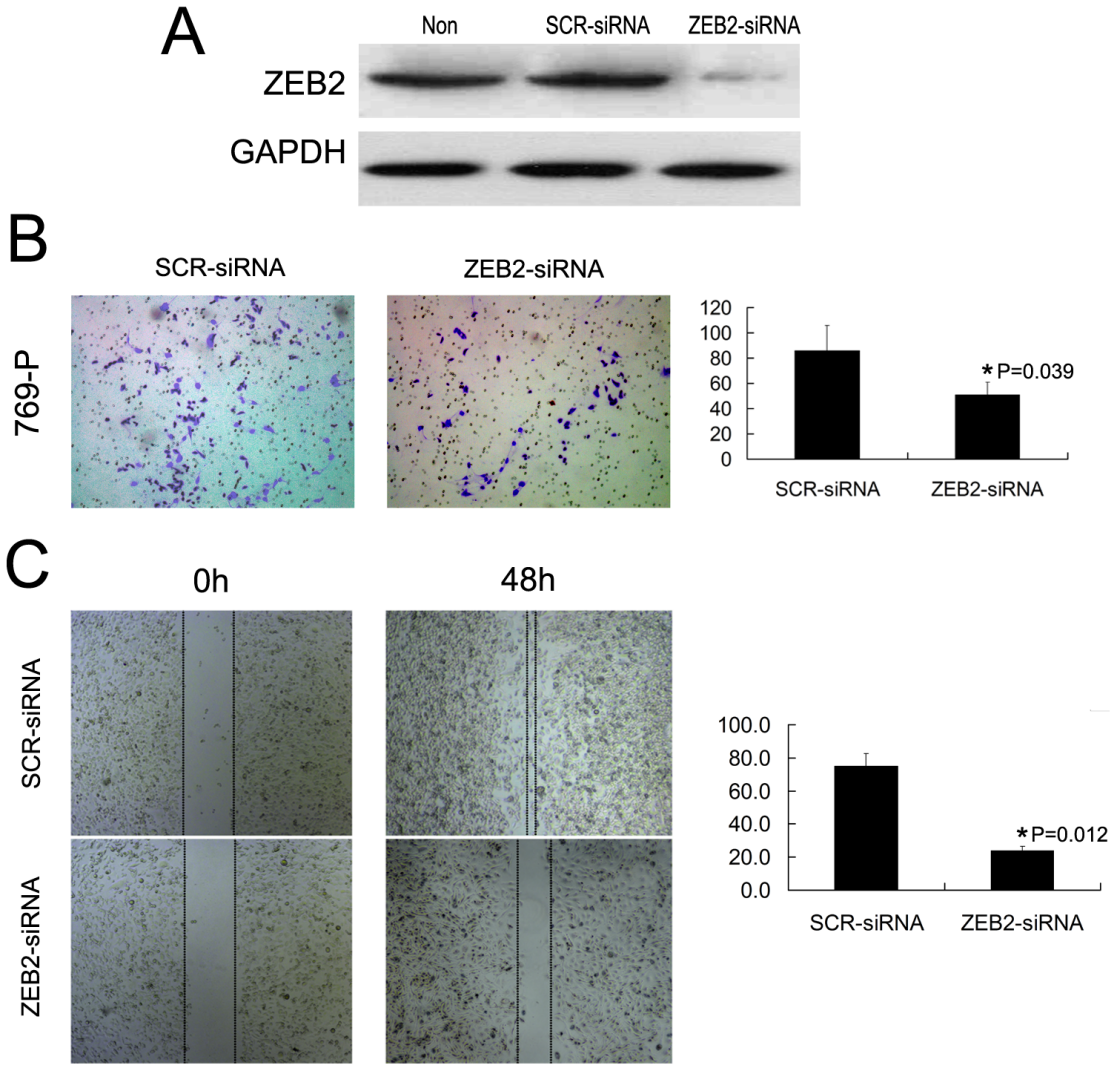
Silencing of ZEB2 by RNA interference inhibits RCC cell migration and invasion. **(A)** Western blotting reveals that ZEB2 was efficiently knocked down by the treatment of ZEB2-shRNA. **(B)** Cell invasion was evaluated using a matrigel invasion chamber. Silencing of ZEB2 decreased 769-P cell invasive capacity. The numbers of invaded cells in siZEB2 and control siSCR groups are shown in the right panel. Error bars indicate ±SE. **(C)** Wound-healing assays show that ZEB2-silenced 769-P cells had lower motility compared with that in control cells. Data are the means±SE of three independent experiments. ^*^ *P*<0.05 by unpaired two-sided T-test.

Because overexpression of ZEB2 in RCC was correlated to an aggressive phenotype in RCC patients, we hypothesized that ZEB2 regulates the migration and/or invasion of RCC cells in vitro. Since 769-P cell showed high expression levels of endogenous ZEB2, ZEB2 was antagonized in vitro by shRNA in this cell line. The transfection and silencing efficiency was evaluated by western blotting and reached approximately 80% and 85%, respectively (Figure 5A). The results of the cell migration and invasion assays demonstrated that the RCC 769-P cell lines transfected with shZEB2 all displayed attenuated migration and invasion abilities compared to the scrambled shRNA control (Figures 5B and 5C).

### Correlations between expression of ZEB2 and EMT markers in RCC

**Figure 6:**
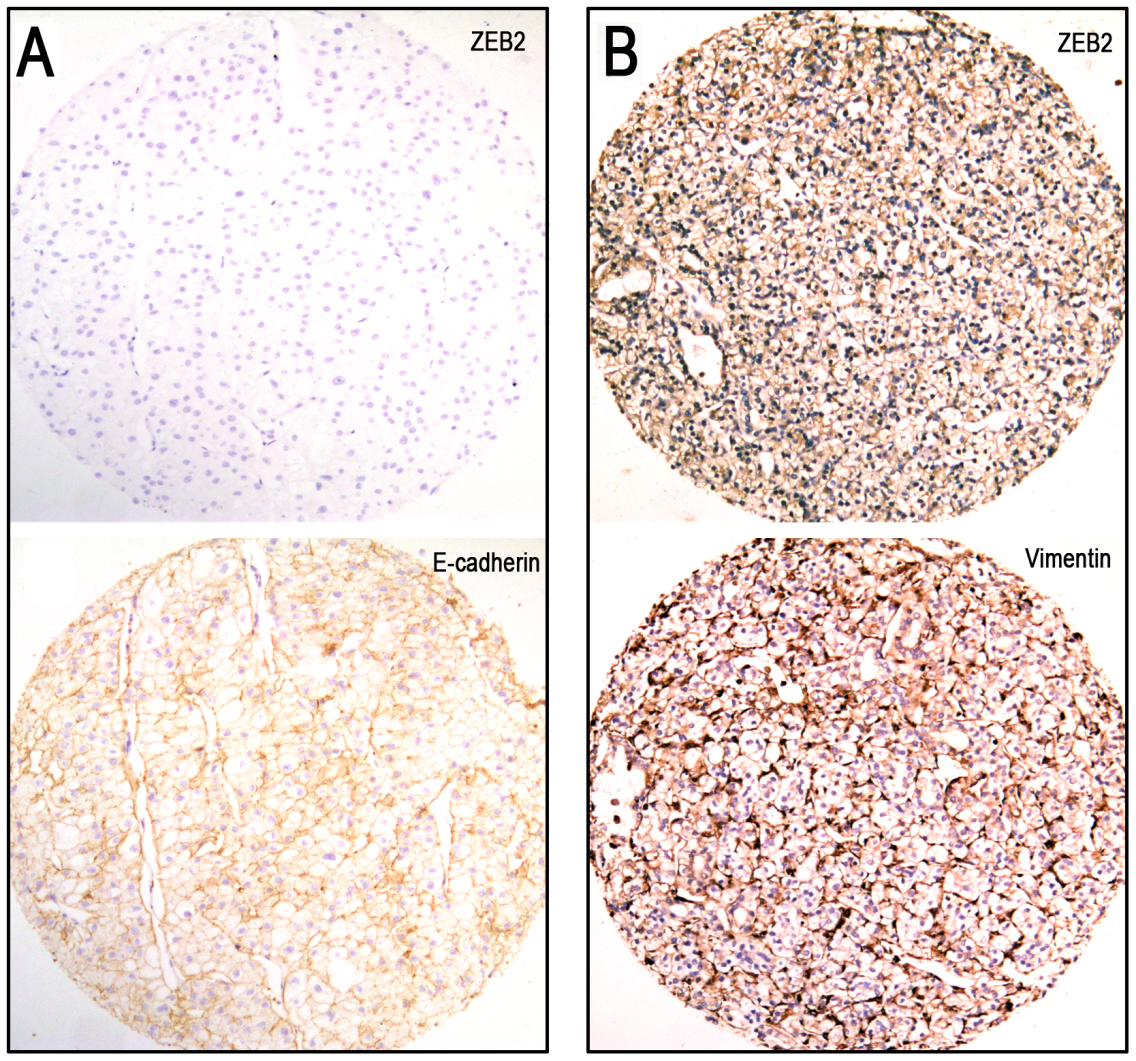
Correlations between ZEB2 expression and expression of E-cadherin or vimentin in RCC tissues. (A) High ZEB2 expression was observed in an RCC (case 87), in which more than 90% of the tumor cells showed positive staining of the ZEB2 protein (*upper panel*, ×100). High vimentin expression was examined in the same RCC case 87 (*lower panel,* ×100). (B) Low ZEB2 expression was shown in an RCC (case 102, *upper panel*, ×100). High E-cadherin expression was observed in the same RCC case 102 (*lower panel,* ×100).

Additional IHC staining of EMT markers (including E-cadherin, β-catenin, vimentin, clusterin and fibronectin) were utilized to analyze the potential correlation between the expression of ZEB2 and EMT markers in RCCs (Figure 6 and Figure S2). Similarly, in our study the cutoff scores for the EMT markers in RCC were determined using a ROC curve analysis. According to the ROC curve, the cutpoints for high E-cadherin, β-catenin, vimentin, clusterin and fibronectin expression were defined when the cases had scores above 45%, 50%, 70%, 65% and 65%, respectively. Further correlation analysis showed that the high expression of ZEB2 was inversely correlated with expression of E-cadherin in our RCC cohort (*P*<0.05, Table 4 and Figure 6A). In addition, a significant positive correlation between ZEB2 expression and vimentin was evaluated in our RCC samples (*P*<0.05, Table 4 and Figure 6B). However, there was no significant correlation between ZEB2 expression and other EMT markers, including β-catenin, clusterin and fibronectin (*P*>0.05, Table 4).

**Table 4:** The correlation between ZEB2 expression and epithelial-mesenchymal transition markers in renal cell carcinoma.

Variable	ZEB2 protein			
	All cases	Low expression	High expression	*P* value*
E-cadherin expression				0.003
Low	133	81 (60.9%)	52 (39.1%)	
High	96	76 (79.2%)	20 (20.8%)	
β-catenin expression				0.282
Low	128	84 (65.6%)	44 (34.4%)	
High	101	73 (72.3%)	28 (27.7%)	
Vimentin expression				0.014
Low	107	82 (76.6%)	25 (23.4%)	
High	122	75 (61.5%)	47 (38.5%)	
Clusterin expression				0.241
Low	140	100 (71.4%)	40 (28.6%)	
High	89	57 (64.0%)	32 (36.0%)	
Fibronectin expression				0.428
Low	112	74 (66.1%)	38 (33.9%)	
High	117	83 (70.9%)	34 (29.1%)	

* Chi-square test.

## Discussion

Despite improvements in surveillance and clinical treatment strategies, the prognosis of RCC remains unsatisfactory because of its high recurrence and distant metastasis rates [4]. At present, the current pTNM stage and pathological grading systems are established and useful prognostic indicators for RCC. However, patients with the same clinical stage and/or pathological grade of RCC often demonstrate considerable variability in disease recurrence and metastasis. Thus, there is a need for new objective strategies that can effectively distinguish between patients with favorable or unfavorable prognoses in the same stage and/or grade. Although RCC has been widely studied, the search for and identification of promising molecular and/or genetic alterations in RCC cells with clinical/prognostic significance remains substantially limited.

ZEB2 is part of the ZEB family of transcriptional factors. These transcription factors contain a central homeodomain, CtBP-binding and Smad-interacting domains and two zinc finger clusters [10], [11]. ZEB2 has been found to mediate the EMT and disease aggressiveness in various human cancers [16], [24]. Previous reports also demonstrated increased levels of ZEB2 transcripts in association with invasion and metastasis in advanced stage cancers [15], [25], [26]. However, expression of the ZEB2 protein in RCC and its clinicopathologic/prognostic significance in RCC are still unclear. Therefore, we employed western blot, high-throughput TMA and IHC to investigate the expression status of ZEB2 in RCC tissues and its significance in patient survival.

In this study, our data showed that the IHC staining of ZEB2 in RCC samples displayed cytoplasmic and/or nuclear localization patterns. Similar results were also found in esophageal, gastric, colorectal and ovarian cancers [27], [28]. The ZEB2 antibody was also used in the Human Protein Atlas (HPA) study (http://www.proteinatlas.org/ENSG00000169554/cancer/renalcancer), and the expression pattern of ZEB2 was different from that obtained in our study. In HPA study, 4/11 (36.4%) of RCC samples showed positive expression of ZEB2, and the immunoreactivity was mainly observed in the nuclei of the tumor cells. However, it has been already reported that the overexpression of ZEB2 is observed in most RCC samples [28]. Consistent with our finding, a recent study also showed that the strong cytoplasmic expression of ZEB2 could be detected in normal epithelial cells, including hepatocytes, kidney tubules, stomach glandular and colon surface epithelium. Furthermore, nuclear translocation of ZEB2 appeared to be prevented in these tissues [28]. Our western blot results revealed that expression levels of the ZEB2 protein in RCC tissues were significantly higher than the corresponding adjacent renal tissues. A previous study showed that elevated ZEB2 transcripts were detected in von Hippel-Lindau-null renal cell carcinomas in a hypoxia-inducible factor 1 alpha-dependent manner [18]. Additionally, Harada et al. [29] indicated that the expression of ZEB2 was significantly associated with the histological subtype of RCC but not with RCC cancer recurrence. However, in our study, high ZEB2 expression in RCC tissues was found to positively correlate with the TNM stage in the validation cohort. Additionally, this was closely correlated with the patient overall survival and progression-free survival. The different methodology used in the IHC evaluation, the small sample size and tumor heterogeneity may contribute to these discrepant findings. Cai et al. utilized ROC curve analysis to determine the cut-off score for ZEB2 expression and found that an H score of 70 in the tumor tissue was the cutpoint for ZEB2 in hepatocellular carcinoma [20]. To avoid using a predetermined and arbitrary set cutpoint, a ROC curve analysis was also used to determine the cut-off score for high ZEB2 expression in our study.

The prognostic significance of ZEB2 expression in RCC patients is the most important finding of the current study. We found that the high expression of ZEB2 was a strong and independent predictor of shortened overall survival, as evidenced by univariate and multivariate analyses. Importantly, a stratified survival analysis of RCC according to the clinical stage and Fuhrman grade showed that ZEB2 expression was closely correlated with RCC patient survival. Our data suggest that ZEB2 expression in RCC may facilitate an increased malignant feature and/or a worse prognosis in this tumor type. Similar results were also observed in breast, ovarian, kidney, oral and bladder cancers [15], [16], [18], [30]. For instance, ZEB2 overexpression also caused resistance to DNA damage-induced apoptosis and correlated with a poor outcome in bladder cancer patients [15]. In addition, shRNA-mediated ZEB2 knockdown significantly inhibited the ability of either cell migration or cell invasion of 769-P cells. Thus, the IHC examination of ZEB2 expression could be used as an additional tool to identify those RCC patients at risk of malignant progression. Our results do not provide a more profound mechanistic study for our observations. However, the ZEB2 expression analysis may also be useful in optimizing individual RCC therapy management, favoring a more aggressive regimen in tumors with high ZEB2 expression. Moreover, ZEB2 might be a new target for anticancer therapy because it plays a critical role in cancer cells.

With regards to the role of ZEB2 in different human cancer types, some of the reports are totally contradictory. ZEB2 has been thoroughly investigated for its role in repression of E-cadherin expression, which is a central event in the EMT [24]. Additionally, ZEB2 upregulation of the EMT and tumor invasion-related genes, such as E-cadherin, vimentin, and metalloproteases, have been reported [15]. However, ZEB2 was shown to inhibit expression of cyclin D1 and was partly responsible for hTERT repression [31], [32]. Other studies described posttranscriptional regulation mechanisms, such as those mediated by the miR-200 family and ZEB2 NAT, in the downregulation of ZEB2 in different pathophysiological contexts [33], [34], [35], [36]. In the present study, we observed that overexpressed ZEB2 correlated with decreased E-cadherin expression and vimentin overexpression in our RCC cohort. Taken together, the previous and present results suggest that ZEB2 may contribute to the development of metastasis through EMT promotion, which may occur not only by downregulating E-cadherin but also by upregulating mesenchymal genes, such as vimentin. Additionally, there was no significant association between ZEB2 expression and other EMT markers (including β-catenin, clusterin and fibronectin). However, additional studies are required to fully understand the underlying function of ZEB2 and the mechanisms that regulate E-cadherin and vimentin expression.

In brief, our findings provide a basis for the concept that high ZEB2 expression may represent an acquired metastatic phenotype of RCC. More importantly, our study introduces high ZEB2 expression as a new adverse independent prognostic factor in RCC. This latter finding is potentially significant because it may help us target a subset of the RCC patient population for more aggressive postsurgical adjuvant anticancer therapies.

## Supporting Information

Figure S1 **The expression dynamics of ZEB2 in renal cell carcinoma.** The 10 images showed the expression of ZEB2 protein by 10% increments in RCC cases.
(TIF)

Figure S2 **The expression patterns of EMT markers** **in RCC tissues by immunohistochemistry.** High β-catenin, clusterin and fibronectin expression were shown in representative cases of RCC patient samples.
(TIF)

Table S1 **The dynamics of ZEB2 expression in renal cell carcinoma.**
(DOC)
