# Supplementary figures and images for: Protein Expression of ZEB2 in Renal Cell Carcinoma and Its Prognostic Significance in Patient Survival
Source: PLoS One. 2013 May 2;8(5):e62558. doi: 10.1371/journal.pone.0062558 (PMC3642198; doi:10.1371/journal.pone.0062558)

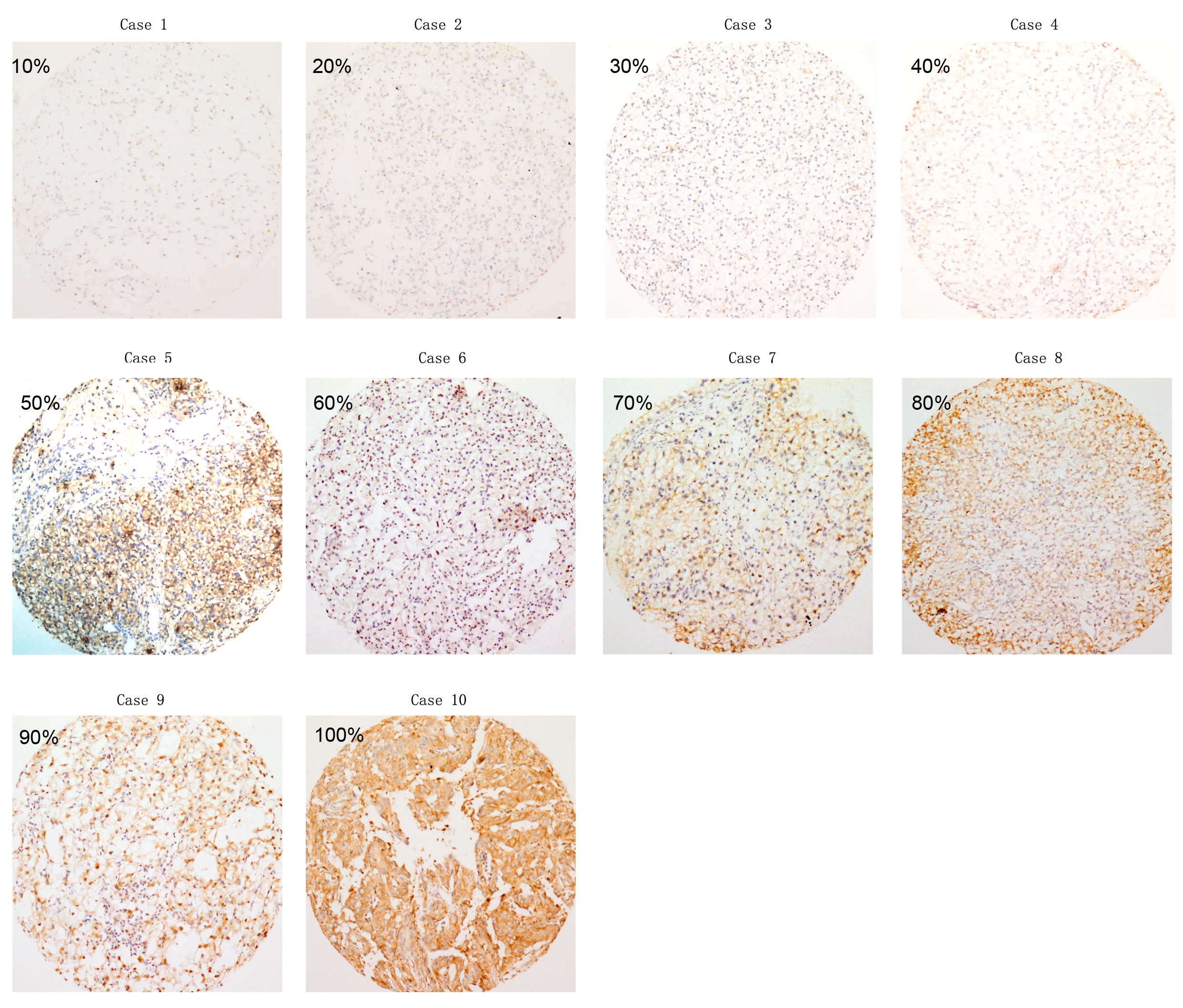

Supplement: Figure S1 — The expression dynamics of ZEB2 in renal cell carcinoma. The 10 images showed the expression of ZEB2 protein by 10% increments in RCC cases. (TIF) [file pone.0062558.s001.tif]

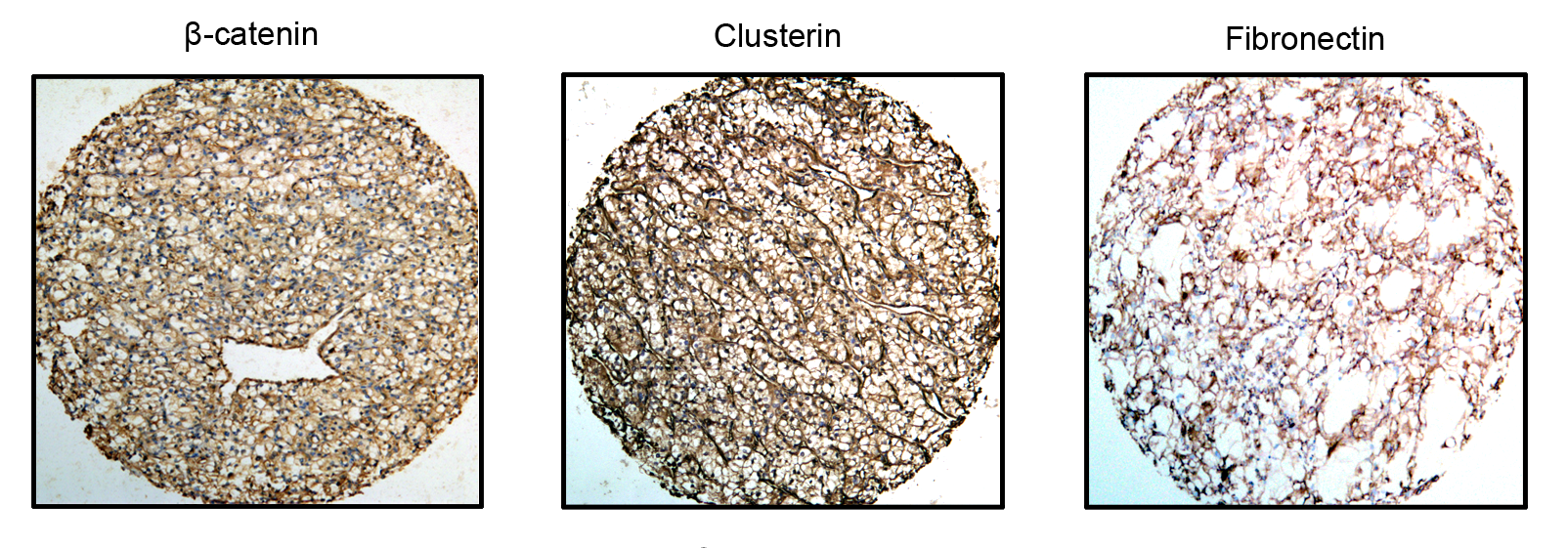

Supplement: Figure S2 — The expression patterns of EMT markers in RCC tissues by immunohistochemistry. High β-catenin, clusterin and fibronectin expression were shown in representative cases of RCC patient samples. (TIF) [file pone.0062558.s002.tif]
