# Supplementary material for: Protein Expression of ZEB2 in Renal Cell Carcinoma and Its Prognostic Significance in Patient Survival
Source: PLoS One. 2013 May 2;8(5):e62558. doi: 10.1371/journal.pone.0062558 (PMC3642198; doi:10.1371/journal.pone.0062558)
Supplement: Table S1 — The dynamics of ZEB2 expression in renal cell carcinoma. (DOC) [file pone.0062558.s003.doc]

**Supporting Table. The dynamics of ZEB2 expression in renal cell carcinoma**

| **ZEB2 expression** | **Number** |
| --- | --- |
| **Low expression (%)** |  |
| 0 | 43 |
| 5 | 8 |
| 10 | 14 |
| 15 | 13 |
| 20 | 14 |
| 25 | 13 |
| 30 | 10 |
| 35 | 15 |
| 40 | 11 |
| 45 | 6 |
| 50 | 8 |
| 55 | 2 |
| Mean % +/- Std. dev | 19.27 ± 16.63 |
| **High expression (%)** |  |
| 60 | 17 |
| 65 | 5 |
| 70 | 15 |
| 75 | 5 |
| 80 | 12 |
| 85 | 3 |
| 90 | 8 |
| 95 | 3 |
| 100 | 4 |
| Mean % +/-Std. dev | 74.86 ± 12.33 |
